# Conference report: Introducing oncology to undergraduate medical and allied health sciences students: reflections from 2nd *e*cancer TMC Oncology Congress 2023 at Kolkata, India

**DOI:** 10.3332/ecancer.2023.1647

**Published:** 2023-12-15

**Authors:** Soumitra Shankar Datta, Sanjit Agrawal, Prateek Jain, Jeevan Kumar, Arnab Bhattacharjee, Ayush Bansal, Shagun Mahajan, Dibakar Podder, Kapila Manikantan, Gaurav Kumar, Bidisha Samanta, Sohini Sarkar, Soumita Ghose, Niladri Ghosal, Mary Guevera, Danny Burke

**Affiliations:** 1 Department of Palliative Care and Psycho-Oncology, Tata Medical Center, Kolkata 700160, India; 2 Honorary Researcher, Institute of Clinical Trials and Methodology, University College London, London WC1E 6BT, UK; 3 Department of Breast Surgery, Tata Medical Center, Kolkata 700160, India; 4 Department of Head Neck Surgery, Tata Medical Center, Kolkata 700160, India; 5 Department of Clinical Haematology and Cellular Therapeutics, Tata Medical Center, Kolkata 700160, India; 6 Department of Medical Oncology, Tata Medical Center, Kolkata 700160, India; 7 Department of Medical Administration, Tata Medical Center, Kolkata 700160, India; 8 Department of Clinical Oncology, North Wales Cancer Center, Rhyl LL18 5UJ, UK; 9 ecancer, 13 King Square Avenue, Bristol BS2 8HU, UK; a https://orcid.org/0000-0003-1674-5093

**Keywords:** oncology, training, undergraduate students, LMIC

## Abstract

Despite the high cancer burden in low-middle-income-countries, medical students often have inadequate exposure to oncology. This may contribute to reduced interest in pursuing training in the field. The second ecancer TMC Oncology Congress at Kolkata on 30th September and 1st October 2023 was planned primarily to introduce undergraduate medical and allied health science students to oncology. There were separate sessions on breast cancer, thyroid cancer, myeloma and research methods so that students get exposure to a wide range of topics. Multi-disciplinary case-based discussions on common clinical presentations helped the students grasp the way a modern cancer hospital functions. Eighty-two percent (131/159, 82%) of the pre-registered delegates attended the congress alongside 44 national and international faculty from surgical oncology, radiation oncology, medical oncology, nuclear medicine, radiology, histopathology, psychiatry and palliative medicine. Of those who offered written anonymous feedback, 76% (70/91, 76%) rated the congress to be excellent. Broadly the following themes emerged from the qualitative feedback a) Delegates positively viewed the opportunity to ‘interact and learn from some of the best of minds in the field of medicine’ b) Suggestions included ‘more interactive sessions through case histories, demonstrations of techniques, videos, quizzes, etc.’ to make the learning experience more engaging. c) Considerable appreciation was expressed for learning about ‘scientific writing’ d) A few delegates were also inspired by the ‘style’ of some of the presentations and felt that this would help to design their presentations in the future. Introducing oncology early during their career may inspire undergraduate students to explore the option of pursuing a career in oncology and allied specialties. A video summarising the event is available at https://ecancer.org/en/video/11672-introducing-oncology-to-undergraduate-medical-and-allied-health-sciences-students. All the talks presented during the conference are available at https://ecancer.org/en/conference/1505-2nd-ecancer-tmc-kolkata-oncology-congress.

## Introduction

Over the years, there have been continued concerns about inadequate exposure to oncology during undergraduate training leading to reduced interest in pursuit of training in oncology and related specialties [1]. For postgraduate students in oncology, there is a more organised curriculum across subspecialties. However, a recent review by Giuliani *et al* [2] on the global oncology curriculum pointed out that the majority of oncology teaching is based on Western ideals. This review [2] mentioned that this may have had an unintended consequence of ‘neo-colonisation’ of curricular contents based on Western medical values and practices. The majority of the global cancer burden is from Low-middle-income-countries (LMICs) [3] and we believe the curriculum for teaching oncology and allied specialties has to be contextualised to the setting where the future specialists will operate while retaining the scientific principles developed in any part of the world. *e*cancer, a UK registered charity, has been publishing an open-access journal in the field of oncology (*e*cancermedicalscience) that is widely accessed from around the globe. The journal formalised its editorial policy of having at least one author for most of its publications from an LMIC. This policy move has been reflected in the recent data from the journal. In the preceding year of 2022–2023, 99% of the papers accepted for publication by *e*cancermedicalscience have at least one author from an LMIC [4]. Alongside academic publications, to bridge the gap in global oncology training, *e*cancer has regularly collaborated and brought together local cancer specialists and international experts while conducting its face-to-face oncology meetings in resource-poor countries of Latin America, Africa and Asia. The current report summarises the learning that happened during the organisation of the 2nd *e*cancer TMC Oncology congress in Kolkata on 30th September–1st October 2023.

## Session on management of breast cancer (day 1 morning)

The session was opened by Dr Sanjit Kumar Agrawal (organising secretory, Senior Consultant, Breast Oncosurgery, Tata Medical Center, Kolkata, India) with a welcome note. He introduced the agenda to the delegates.

The talks were mainly focused on the basics of breast surgery and multimodality management of breast cancer. The various topics that were covered are as follows.

### How to approach a breast lump?

This talk delivered by Professor Anand Mishra, KGMC Lucknow, India, emphasised that a triple assessment is to be done for all suitable patients. Professor Mishra explained the diagnostic pathway of breast cancer detection to the delegates.

### American Joint Cancer Control (AJCC) staging of breast cancer

This talk was delivered by Professor S.V.S Deo, AIIMS, New Delhi, India. The crucial role of the biological subtype of breast cancers and AJCC 8th breast cancer staging was discussed. He explained the role of neoadjuvant therapy in triple-negative and HER 2-positive patients and the importance of chemo-response-adapted therapy.

### Breast cancer screening in LMICs

Professor Diptendra Sarkar, IPGMER & SSKM Hospital, Kolkata, India discussed breast cancer screening roadmap for developing countries like India. He also explained the evidence base for low-cost clinical breast examination as a screening tool.

### Surgical steps of modified radical mastectomy

Dr Abhishek Shrama, Consultant, Breast Oncosurgery, TMC, Kolkata, India introduced the audience to the technique of modified radical mastectomy.

### Breast conservation surgery

Dr Neha Choudhary, Consultant, Breast Oncosurgery from Narayana Superspeciality Hospital, Howrah, India covered the surgical techniques of breast conservation surgery with case examples and video demonstrations.

### Axillary management in breast cancer

This talk by Dr Sanjit Kumar Agrawal, Senior consultant, Breast Oncosurgery, TMC, Kolkata, India discussed in detail about the importance of axilla preservation in non-screened breast cancer patients by offering sentinel lymph node biopsy to all eligible patients.

### Panel discussions on management of breast cancers

A series of panel discussions followed the talks. The panel discussions were on multimodality management of early, advanced and metastatic breast cancer. Moderators presented anonymised case histories and examination findings of past patients with breast cancer (anonymised and de-identified). Various nuances of management of these patients were discussed with panelists consisting of breast surgeons, medical oncologists, radiologists, radiation oncologists and palliative care experts.

## Session on management of multiple myeloma (day 1 afternoon)

Multiple myeloma is a cancer of plasma cells, which is a type of white blood cell. Monoclonal plasma cells proliferate in bone marrow, resulting in an increase in monoclonal paraprotein (M protein), destruction of bone, and displacement of other hematopoietic cells. The second session of the 2nd *e*cancer Oncology Congress 2023 was on the assessment and management of multiple myeloma for undergraduate and postgraduate medical students. Common symptoms of multiple myeloma may include weight loss, bone pain (often backache), fractures, weakness, or fatigue. Diagnosis of myeloma requires multiple investigations like complete blood counts, serum calcium, serum creatinine, serum and urine assessment for monoclonal protein, bone marrow aspiration and biopsy and imaging tests. The two talks on multiple myeloma were as follows.

### Myeloma: when to suspect and how to diagnose?

The first talk ‘Myeloma: when to suspect and how to diagnose?’ was delivered by Dr Bishesh Boudel, Professor of Clinical Haematology, Civil Hospital, Kathmandu, Nepal. This talk made the students aware of the common and unusual symptoms with which patients with myeloma can present for the first time and the ways to assess these patients. He discussed how the results of the diagnostic workup determine the initial classification of the patient's illness into monoclonal gammopathy of undetermined significance (MGUS), solitary plasmacytoma, smoldering multiple myeloma, or symptomatic multiple myeloma.

### Treatment and prognosis of myeloma

Professor Maitreyyee Bhattacharyya from the Medical College, Kolkata, India spoke on the treatment and prognosis of myeloma. MGUS often does not need active treatment but requires very close monitoring. Radiation therapy is the primary therapy for solitary plasmacytoma. Treatment of multiple myeloma often consist of a combination of targeted therapy, chemotherapy, radiation therapy and stem cell transplantation.

## Session on management of thyroid cancers (day 1 afternoon)

The head and neck session in the workshop dealt with thyroid malignancies. Thyroid malignancy is a very common malignancy in India with an age-standardised incidence of 3.5/100,000 for women and 1.3/100,000 for men [5]. The sessions broadly covered the diagnosis, management and recent controversies on the management of thyroid cancer over several lectures and panel discussions.

### Diagnosis in thyroid lesions

#### Bethesda and beyond – improving results of FNAC in thyroid nodules

In the first talk, Dr Indu Arun from Tata Medical Center, Kolkata, India touched upon the details of the Bethesda system and the way forward. This was done in a way such that it is understandable to medical students and young clinicians.

#### Imaging and intervention in thyroid lesions

Dr Dayananda Lingegowda from Tata Medical Center, Kolkata, India gave details about various imaging modalities for evaluating the thyroid and shared his experience about the role of radiofrequency ablation in thyroid lesions.

### Treatment of thyroid lesions

#### Remote access thyroid surgery

During his talk, Dr Rajeev Sharan from HCG EKO Cancer Centre, Kolkata, India explained to the students the current practices in the management of thyroid malignancies, including recent advances in remote access thyroidectomy. The delegates could get a glimpse of the technique of remote access thyroid surgery through a demonstrative operative video presented by Dr Sharan. Any surgery can be associated with complications and prevention and management of these complications was also discussed.

#### Management of surgical complications in thyroid surgery

Dr Kapila Manikantan (Tata Medical Center, Kolkata, India) in his talk on complications of thyroidectomies, stressed the importance of precise knowledge of anatomy, leading to the prevention of complications in thyroid surgeries.

#### Looking at radioiodine and other radio-labelled metabolites for thyroid cancers

The role of different radionucleotides in the diagnosis and treatment of thyroid malignancies was dealt with by Dr Jayanta Das, Consultant, Department of Nuclear Medicine, Tata Medical Center, Kolkata, India.

### Panel discussion

The panel discussion was directed at controversies in the management of thyroid malignancies and included discussions on both diagnostic and therapeutic aspects of thyroid malignancies. Through actual case summaries seen in the clinic, the panel discussed nuances of the management of thyroid malignancies in both the early and late stages of the disease.

All the speakers for the session on thyroid cancers structured their presentations keeping in mind the younger audience of the conference. The lectures were well received by the delegates and generated a lot of questions that were discussed at the end of each talk. The speakers believed that this conference would have helped a few of the delegates to consider head and neck oncology as a potential career path.

## Session on research methods in oncology (day 2, morning)

### Interpreting the results of a clinical trial moderator

Critically appraising a randomised control trial lays the foundation for applying evidence-based medicine to day-to-day clinical practice and this was covered by Dr Sanjit Agrawal in the opening talk of the second day of the 2nd *e*cancer TMC Oncology Congress 2023, Kolkata.

### Writing IMRAD: introduction, methods, results, discussion

Through this talk Dr Soumitra Shankar Datta (Tata Medical Center, Kolkata, India), Organising Chair for the conference tried to inspire young clinicians and researchers to start writing research papers. This talk touched upon content, structure, language, style and rhetoric, all of which are crucial in writing various different sections of a research paper, such as ‘introduction’, ‘methods’, ‘results’, ‘discussions’ and ‘abstract’. The talk also covered ways to respond to peer reviewer’s comments.

## McVie-Veronesi award session (day 2 morning)

The final and last session of the conference had seven presentations from various participants whose research papers were selected for the session through a competitive process. This session was chaired and judged jointly by Professor Bishesh Poudel, Professor of Clinical Haematology, Civil Hospital, Kathmandu, Nepal and Dr Prateek Jain, Consultant Head Neck Surgeon, Tata Medical Center, Kolkata, India. Ms. Soumita Ghose from Tata Medical Center, Kolkata won the first prize for her presentation titled ‘Insights into Journeys of Cancer Patients in India – Access, Affordability and Barriers’. The second prize was won by Dr Srijan Das from the Central Institute of Psychiatry, Ranchi, India who presented on ‘Harnessing Artificial Intelligence to Mitigate Depression Rates in Cancer Clinicians’. The 3rd prize winner was Dr Akella Phanendra from Indo-American Cancer Hospital, Hyderabad, India, who presented his findings from a research study by his team titled ‘Role of Sentinel Lymph Node Biopsy In Node-Positive Breast Cancer Patients’ All seven research presentations were of excellent quality.

## Delegates, faculty and their feedback

**Figure 1.**
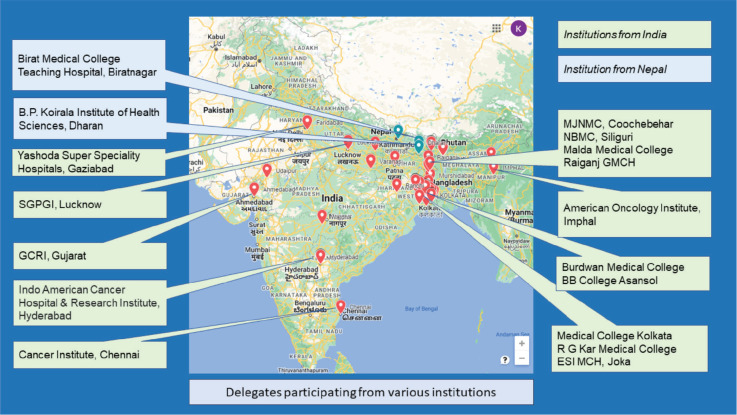
Delegates participating from various institutions from India and Nepal.

Eighty-two percent of the delegates who pre-registered (131/159, 82%) for the conference had attended the oncology congress. The delegates had come from a wide geographical region of India and Nepal (Figure 1) and a majority were undergraduate students and young professionals (Table 1).

In addition to the 131 delegates, there were 45 national and international faculty from various specialties of oncology as Surgical Oncology, Medical Oncology, Radiation Oncology, Clinical Haematology, Radiology, Histo-pathology, Nuclear Medicine, Palliative Medicine and Psychiatry.

The didactic teaching presentations, case discussions in a multi-disciplinary format and opportunities for undergraduate and postgraduate students to present their research provided a foundation for academic interactions between the faculty and delegates. Anonymous feedback was collected from the majority of the delegates (*n* = 91) after the completion of the second day of the conference. Some items of the feedback questionnaire had options of being marked on a 4-point Likert scale and a few items welcomed free text responses. Most of the participants (70/91, 76.93%) rated the overall experience of the conference to be ‘excellent’. While delegates were happy with the content and pace of training, they wanted the inclusion of more interactive sessions and hands-on activities. Around 79% of participants (72/91, 79.1%) felt that 'they had learned something useful' and about 87% of participants (79/91, 87%) ‘were glad that they had come’ for the event (Table 2). Fifteen delegates (15/131, 11.4%) won *‘Professor Gordon McVie Travel Bursary’* to attend the conference through a formal competitive application process. Overall, a very low registration fee of Rs 500 (around 6 dollars) ensured that the event was affordable to all undergraduate and post graduate students. A few of the travel bursary winners, mentioned in their feedback that the travel bursary partly supported their expenses to attend the conference.

The free text portion of the feedback asked participants what they liked about the congress and how they would want future sessions to be organised. Broadly the following themes emerged from the qualitative feedback. a) Delegates positively viewed the opportunity to ‘interact and learn from some of the best of minds in the field of medicine’. b) Suggestions included ‘more interactive sessions through case histories, demonstrations of techniques, videos, quizzes etc.’ to make the learning experience more engaging. c) Considerable appreciation was expressed for learning about ‘scientific writing’. d) A few delegates were also inspired by the ‘style’ of some of the presentations and felt that this would help to design their presentations in the future (Table 3).

The conference received a warm and enthusiastic response from participants (Video 1). The faculty felt the interaction was meaningful as relayed by senior oncologists of the country (Video 2 and 3).

## Conclusion

Following the 2-day oncology congress, the feedback from delegates showed that introducing oncology to undergraduate and postgraduate medical and allied health science students was welcomed by the students. In a country like India that has a high cancer burden, it is important to inspire young healthcare professionals early in their course of training to consider taking up oncology and allied specialties as future career options. Having a mix of didactic teaching alongside case discussions and research presentations by the delegates can make teaching events participatory and more meaningful. Following on from the feedback received, in future we may further explore to include a ‘peer led education’ session engaging credible postgraduate students working in oncology and allied specialties to facilitate learning of under-graduate students. Having breakout rooms and interactions in smaller teams may also foster engagement of younger participants. More research is needed on teaching styles that introduce undergraduate students to oncology. Introducing oncology early during their career may inspire undergraduate students to explore the option of pursuing a career in oncology and allied specialties.

## Conflicts of interest

The authors declared they have no competing interests.

## Funding

The 2nd *e*cancer TMC Oncology Congress 2023 at Kolkata, India was funded by *e*cancer. The open access publication charges of this paper are also supported by *e*cancer (UK Charity number 1176307).

## Figures and Tables

**Video 1.**
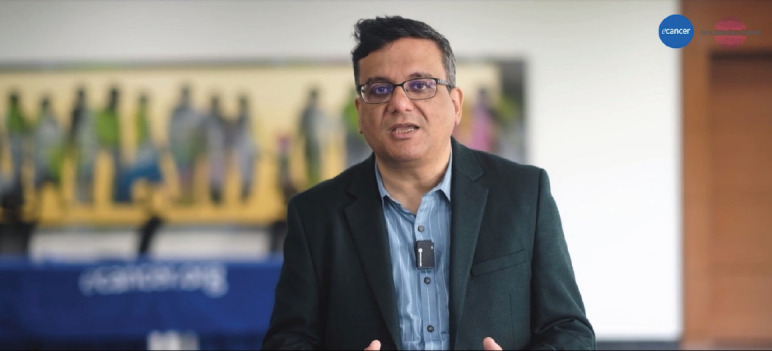
Feedback and reflections on the conference from the Organising Chair Dr Soumitra Shankar Datta. To view this video, click here: https://ecancer.org/en/video/11672-introducing-oncology-to-undergraduate-medical-and-allied-health-sciences-students.

**Video 2.**
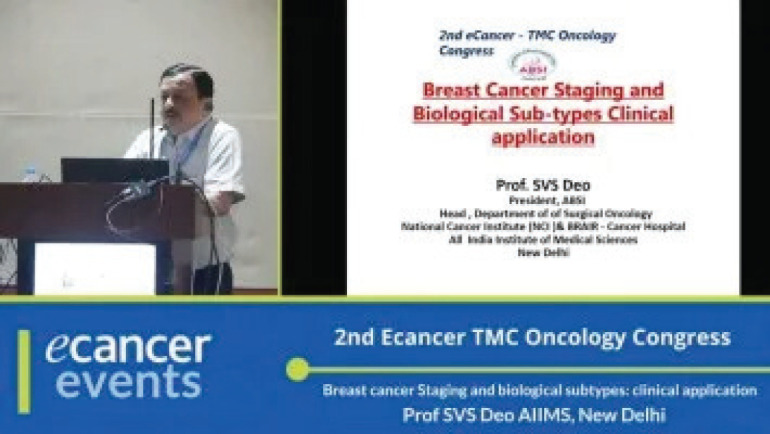
Reflections from Professor SVS Deo, Professor and Head of Surgical Oncology, All India Institute of Medical Sciences, New Delhi. To view this video, click here: https://ecancer.org/en/video/11542-breast-cancer-staging-and-biological-subtypes-clinical-application.

**Video 3.**
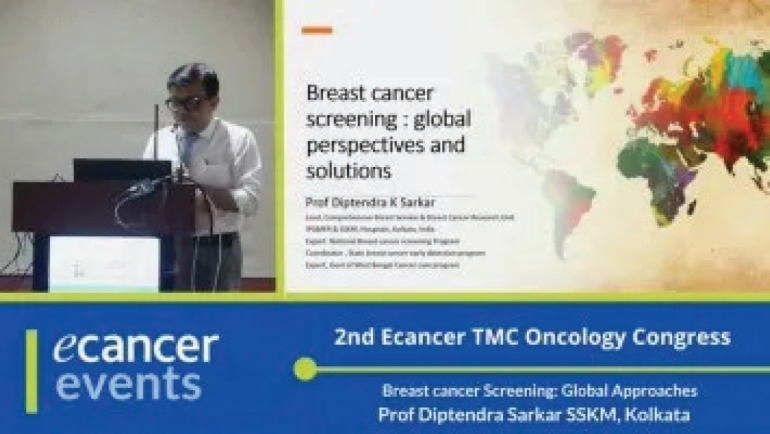
Professor Diptendra Sarkar, Professor of Surgery, Comprehensive Breast Service, Department of Surgery, Institute of Post Graduate Medical Education and Research and SSKM Hospitals, Kolkata. To view this video, click here: https://ecancer.org/en/video/11538-breast-cancer-screening-global-approaches.

**Table 1.** Demographic and educational background of delegates who attended the congress.

Variable		*N* = 131	%
Age range	16-21 years	45	34.35
	22-30 years	66	50.38
	31-40 years	16	12.21
	41-50 years	3	2.29
	51-60 years	1	0.76
Gender	Male	84	64.12
	Female	47	35.87
Specialty	Medical student (MBBS)	69	52.67
	Postgraduate medical student (MD/MS/DNB)	21	16.03
	DM/MCh/Super-speciality student	2	1.52
	Professional (Senior resident)	5	3.81
	Junior faculty	4	3.05
	Allied health professional	30	22.90

**Table 2.** Collated anonymous feedback from delegates after completion of the congress.

Feedback (*n* = 91)	Poor	Average	Good	Excellent
Overall experience	-	-	23.07%	76.93%
Training structure	-	3.22%	24.17%	72.61%
Training content	-	2.19%	21.97%	75.82%
Quality of slides	-	-	21.97%	78.02%
Session length	-	3.29%	37.36%	59.34%
Trainer	-	-	14.28%	85.71%
Venue	1.09%	5.49%	12.08%	81.31%
Pace of training	-	8.79%	38.46%	52.74%
‘I learnt something useful’	-	1.09%	19.78%	79.12%
‘I am glad I came’	-	1.09%	12.08%	86.81%

**Table 3.** Qualitative free-text feedback from delegates.

What do you like about the conference?	How do you think the conference could be made more interesting and improved?	Outline three things that you will take with you/have learned in this conference	Do you have any suggestions for a new session?
*‘The fact that we can have the chance to learn about the experiences of the best possible clinicians from India as well as from around the world was a brilliant addition. The approach towards research-based studies was the best of it all’.*	*‘Some more interactive exercises could be added which would allow the audience to participate’. ‘The research part of the conference could have been more elaborately discussed’.*	*‘1) Interaction with some of the greatest breast cancer doctors in our country. 2) Advancement in thyroid cancer surgery with multimedia presentations with post-operative pictures.*	*‘I would like to suggest providing inclusivity of more videos to aid the ppt-based lectures. E.g.-use surgical clips to teach the breast surgical lectures.*
*‘Great set of highly qualified and experienced doctors came who shared some of their knowledge with us. I liked the pre-conference quiz & basically, that can make things more interactive, like the panel discussion- inspirational’.*	*‘If the conference would include some exercises & interactive sessions, especially with undergraduates like us. We could be closer to the great personalities in the path of medical sciences who were present there’.*	*‘1) Knowledge regarding the most common cancers prevalent in India. 2)Method of approaching a patient’.*	*‘As mentioned in Q3 a quiz post a session of lectures to you know keep everyone on their toes at all points in time throughout the duration of the course. It will be majorly in the form of a game with very basic rewards (Motivation is necessary)’*
*‘This conference offers an excellent opportunity to directly interact with some of the best doctors of the country and learn some of the interesting clinical case study questions from them’.*	*‘Overall the experience was good some points to improve 1) Breaks should be made a bit more frequent 2) The entire session can be made more interactive with quizzes, case presentations, and poster presentations 3) The tone of the conference is pretty serious, the learning can be made more conducive by keeping some fun elements in between’.*	*‘How to approach a patient – starting from the basics of history taking.*	*‘Being a budding radiation oncologist, I would love if more sessions inclusive of techniques and updates in radiotherapy were there’.*
*‘Being a student of second year MBBS, I genuinely got the opportunity of learning a lot of things, that too from the best oncologists & researchers of the country. We had been eager to attend the sessions’.*	*‘1) If the panel sessions could have a briefing (before the congress) about the intricacies that would be helpful. 2) An interim quiz after a prolonged session may help to test the attention span of everyone. (Brownie points for correct answers, the motivation that anybody would want)’.*	*‘1) Mistakes to avoid while writing any scientific literature. 2)To distinguish between factual slides & and engagement slides in the PowerPoint presentations of speakers. I have learned a lot about presenting a session’.*	*‘For new sessions can it be made like a workshop to learn soft skills & not just routine slide presentation’.*
